# Corrosion Products and Microstructural Evolution of Ordinary Portland Cement and High-Performance Concrete After Eight Years of Field Exposure in Qarhan Salt Lake

**DOI:** 10.3390/ma18081769

**Published:** 2025-04-12

**Authors:** Zhiyuan Luo, Hongfa Yu, Haiyan Ma, Yongshan Tan, Chengyou Wu, Jingnan Sun, Xiaoming Wang, Peng Wu

**Affiliations:** 1 School of Civil Engineering, Qinghai University, Xining 810016, China; callmel2022@163.com (Z.L.); wuchengyou86@163.com (C.W.); sunjingnan20220901@163.com (J.S.); xiaoming_wang111@163.com (X.W.); wupeng8686@126.com (P.W.); 2 College of Civil Aviation, Nanjing University of Aeronautics and Astronautics, Nanjing 211106, China; 3 College of Civil Science and Engineering, Yangzhou University, Yangzhou 225127, China; ystan@nuaa.edu.cn

**Keywords:** concrete, salt lake, saline soil, corrosion products, corrosion mechanism, transformation of corrosion products, spatial distribution of corrosion products, long-term durability performance

## Abstract

Salt lakes and the surrounding saline soils distributed across northwestern China and Inner Mongolia impose severe physicochemical corrosion on cement-based concrete. Understanding the corrosion products and mechanisms are crucial scientific and technological factors in ensuring the durability and service life of concrete structures in these regions. In this study, various analytical techniques—including X-ray diffraction, thermogravimetric–differential thermal analysis, X-ray fluorescence, and scanning electron microscopy coupled with energy-dispersive spectroscopy—were employed to systematically analyze the corrosion products of ordinary Portland cement (OPC) and high-performance concrete (HPC) specimens after eight years of field exposure in the Qarhan Salt Lake area of Qinghai. The study provided an in-depth understanding of the physicochemical corrosion mechanisms involved. The results showed that, after eight years of exposure, the corrosion products comprised both physical corrosion products (primarily sodium chloride crystals), and chemical corrosion products (associated with chloride, sulfate, and magnesium salt attacks). A strong correlation could be observed between the chemical corrosion products and the strength grade of the concrete. In C25 OPC, the detected corrosion products included gypsum, monosulfate-type calcium sulfoaluminate (AFm), Friedel’s salt, chloro-ettringite, brucite, magnesium oxychloride hydrate 318, calcium carbonate, potassium chloride, and sodium chloride. In C60 HPC, the identified corrosion products included Kuzel’s salt, Friedel’s salt, chloro-ettringite, brucite, calcium carbonate, potassium chloride, and sodium chloride. Among them, sulfate-induced corrosion led to the formation of gypsum and AFm, whereas chloride-induced corrosion resulted in chloro-ettringite and Friedel’s salt. Magnesium salt corrosion contributed to the formation of brucite and magnesium oxychloride hydrate 318, with Kuzel’s salt emerging as a co-corrosion product of chloride and sulfate attacks. Furthermore, a conversion phenomenon was evident between the sulfate and chloride corrosion products, which was closely linked to the internal chloride ion concentration in the concrete. As the chloride ion concentration increased, the transformation sequence of sulfate corrosion products occurred in the following order: AFm → Kuzel’s salt → Friedel’s salt → chloro-ettringite. There was a gradual increase in chloride ion content within these corrosion products. This investigation into concrete durability in salt-lake ecosystems offers technological guidance for infrastructure development and material specification in hyper-saline environments.

## 1. Introduction

Qarhan Salt Lake, located in the southern part of the Qaidam Basin in western Qinghai Province, China, is the largest salt lake in the country and one of the most renowned inland salt lakes in the world. The region is rich in resources—such as potassium, sodium, magnesium, lithium, boron, bromine, and iodine—making it a valuable catalyst for economic development. With the continuous expansion of salt-lake resource exploitation, many projects have been initiated in this area, including the extensive construction of concrete structures, such as bridges, roads, and industrial facilities [1]. However, the unique geographical and climatic conditions of the Qarhan Salt Lake region pose severe corrosion challenges to concrete structures.

The salinity of salt lake brine in China can reach up to ten times that of seawater [2]. Its main components include chloride, sulfate, and carbonate salts containing potassium, sodium, and magnesium, which can impact concrete structures enormously. The brine of Qarhan Salt Lake consists primarily of chlorides and sulfates [3], whereas the surrounding saline soil is dominated by magnesium chloride, sodium chloride, and potassium chloride as the main chloride salts and by magnesium sulfate and calcium sulfate as the predominant sulfate salts. Corrosion in the salt lake follows a Mg^2+^–Cl^−^–SO_4_^2−^ complex corrosion mechanism, and the surrounding saline soil is classified as chloride-rich saline soil with a strong to extreme degree of salinization.

The ions in the lake brine exert a mutually reinforcing and accelerating effect on concrete corrosion. For instance, chloride ions not only chemically corrode concrete but also contribute to crystallization-induced damage when combined with sodium and potassium ions. A field investigation conducted by Liu Lianxin [1] found that buildings in the salt lake region that lacked specialized protective treatments were quickly corroded, leading to structural failure. Wang Fusheng [4] conducted an extended investigation on structures in salt lake regions, demonstrating that concrete surfaces exposed to saline soil environments typically develop cracks within 1–3 years of exposure. The problem of corrosion failure caused by groundwater brine and saline soil in the salt lake region is severe and widespread. It extends to salt lake environments in the Middle East (e.g., Israel), Africa (e.g., Ethiopia), the Americas (e.g., the United States and Argentina), and Asia (e.g., China).

Calleja [5] examined the corrosion mechanisms affecting concrete in the salt lake environment of the Dead Sea, and analyzed the combined effects of Mg^2+^, Cl^−^, and SO_4_^2−^. Yu Hongfa [6] investigated the corrosion products of Mg^2+^-Cl^−^-SO_4_^2−^ brine. Brown P. W. et al. [7] studied the distribution of corrosion products in concrete under the joint action of Cl^−^ and SO_4_^2−^. Lingyu L [8] investigated the macro-mechanical properties and micro-mechanisms of high-performance concrete after 10 years of immersion in salt lake brine. Long-term research on concrete corrosion in salt lake environments has helped the sustainable development of salt lake resources in northwestern China, as it can significantly reduce construction and maintenance costs. However, the corrosion mechanisms of ordinary Portland cement (OPC) and high-performance concrete (HPC) under long-term field exposure in salt lake environments have remained largely unexplored. Consequently, this study employed modern testing techniques to investigate the corrosion products of OPC and HPC specimens after eight years of field exposure in the Qarhan Salt Lake region and further analyzed the microstructural evolution of the corroded concrete. The findings provide an empirical dataset on long-term corrosion product characteristics of concrete in salt lake regions, while establishing durability performance criteria to inform engineering design and material optimization strategies for infrastructure construction in hyper-saline ecosystems.

## 2. Materials and Methods

### 2.1. Raw Materials and Mix Proportions

The cement used in this study was sourced from Gansu Qilianshan Cement Group Co., Ltd. (Lanzhou, China), and included P.II 52.5 cement (with limestone powder as the active mineral additive) and P.O 42.5 cement. The main physical and mechanical properties of the cement are listed in Table 1. The chemical compositions of the main raw materials are provided in Table 2. The slag used was an S95-grade product from the slag powder plant of Gansu Xiangyang Trading Co., Ltd. (Xiangyang, China). Its main physical and mechanical properties are presented in Table 3, whereas its chemical composition is shown in Table 2.

The fine aggregate was obtained from a sand quarry in Ledu, Qinghai. It consisted of medium sand with a compacted bulk density of 1639 kg/m^3^, fineness modulus of 2.87, and Zone-II-classified gradation. The coarse aggregate was sourced from a quarry in Xiaoxia, Xining. The rock had a compressive strength of 107.3 MPa. For C20–C35 concrete, a maximum aggregate size of 40 mm with a continuous gradation of 5–40 mm was used; for C40–C45 concrete, a maximum aggregate size of 25 mm with a continuous gradation of 5–25 mm was employed; and for C50–C60 specimens, a maximum aggregate size of 15 mm with a continuous gradation of 5–15 mm was selected. The content of the other components is provided in Table 4.

The water reducer was a polycarboxylate superplasticizer produced by Xining Yangjian Waterproof Admixture Co., Ltd. (Xining, China). It was in liquid form, with a water reduction rate exceeding 30%, a chloride-free composition, and a solid content of 37.4%. The use of water conformed to the requirements specified in the JGJ63-2006 standard [9].

This study focused on two strength grades of concrete—C25 and C60 concrete. The C20–C35 range was classified as OPC, whereas the C40–C60 range was designated as HPC. The detailed mix proportions are provided in Table 5.

### 2.2. Concrete Specimens and Field Environment

The field exposure experiment was conducted in the Qarhan Salt Lake region of Qinghai. The surrounding saline soil zone of the salt lake is extensive. Some soils contain fine crystalline salt particles, whereas a few contain salt blocks of 5–8 mm size. Along the route from Golmud to Qarhan, the soil conditions transition as follows: approximately 30 km of mildly saline soil with a salt content of 0.4–4%, approximately 16.5 km of strongly saline soil with a salt content of 3–13%, and approximately 5 km of ultra-strongly saline soil with a salt content from 5 to 44%. The HPC salt lake exposure station of this study is located in the strongly saline soil zone within the Qarhan Salt Lake area of Qinghai Province.

Table 6 presents the chemical composition of the topsoil in the strongly saline soil zone of the Qarhan Salt Lake region. The total salt content reached 37.17%. The Mg^2+^, Cl^−^, SO_4_^2−^, and CO_3_^2−^ ion concentrations were as high as 0.5%, 17.88%, 0.7%, and 4.31%, respectively. The concrete specimens were categorized based on their strength grade and cast into cubic specimens of side length 100 mm. Three specimens were prepared for each strength grade, 24 specimens in total. Indoor curing was conducted following standard procedures. Upon completion of the indoor curing, the specimens were transported to the Qarhan Salt Lake for field exposure testing.

### 2.3. Sampling

After field exposure, the specimens were retrieved and brought back to the laboratory for sampling. Both bulk and powdered samples were collected. The sampling methods were categorized as either direct sampling or core drilling.

#### 2.3.1. Direct Sampling

**Figure 1:**
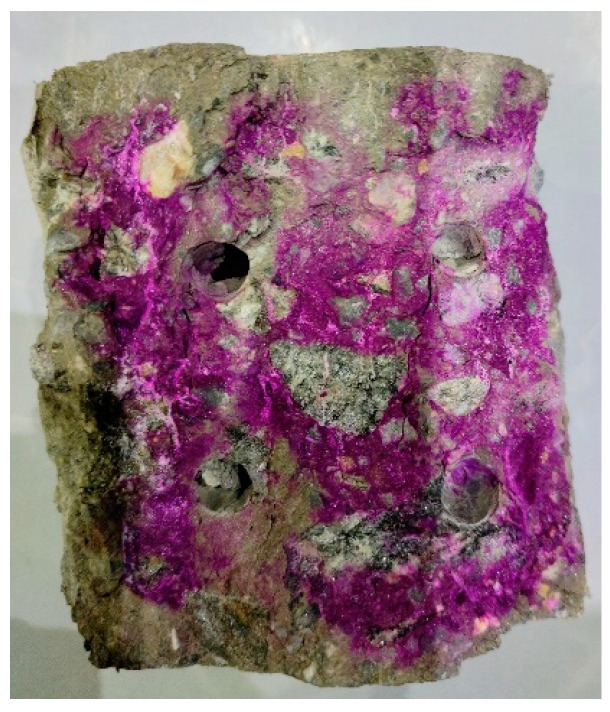
Cross-sectional view of a specimen subjected to direct sampling.

Prior to direct sampling of the mortar, the specimens had undergone compressive strength testing and carbonation depth testing. Following these tests, samples were taken from the failure surfaces of the specimens, specifically from regions that remained uncolored in the carbonation depth tests, which is shown in Figure 1. The thickness of the sampled uncolored surface layer ranged from 0 to 5 mm, and the directly obtained samples were in bulk form.

#### 2.3.2. Core Drilling Sampling

Core drilling sampling involved the extraction of a total of eight samples from two opposing surfaces of each specimen. The drilling locations were 20 mm from the surface edges, with four drill holes per surface. Each drill hole was incrementally drilled to a depth of 5 mm per step, reaching a total depth of 40 mm. The obtained powdered samples were divided into eight groups, corresponding to each 5 mm depth increment. Owing to severe failure, C20 specimens were not suitable for collecting powdered samples via the core drilling process. In some specimens, unexpected circumstances arose during the drilling process, leading to the absence of samples from certain depth intervals.

### 2.4. Testing Methods

#### 2.4.1. X-Ray Diffraction (XRD) Testing of Samples

X-ray diffraction (XRD) analysis was performed using a Rigaku D/max-2500PC X-ray diffractometer (Tokyo, Japan) with a CuKα radiation source, accelerating voltage of 40 kV, scanning range of 5–70°, and scanning speed of 6°/min. The test samples included surface bulk samples obtained via direct sampling, which were ground and sieved to meet the 200-mesh fineness requirement, as well as powdered samples acquired through core drilling. The latter were mixed with 20% by mass of α-alumina and ground for 10 min to prepare the test specimens.

#### 2.4.2. Thermal Analysis of Samples

Thermogravimetric–differential thermal analysis (TG-DTA) was conducted using a Type 1 thermal analyzer manufactured by Beijing Hengjiu Experimental Equipment Co., Ltd. (Beijing, China). The temperature was increased at a rate of 10 °C/min, starting from 25 °C and terminating at 1100 °C. The test samples were prepared by grinding the bulk samples obtained via direct sampling into a powdered form.

#### 2.4.3. X-Ray Fluorescence (XRF) Analysis of Samples

X-ray fluorescence (XRF) analysis was performed using an X-ray fluorescence spectrometer produced by Rigaku (Akishima, Japan). The instrument was equipped with an end-window Rh target X-ray tube, operating at a rated voltage of 4 kW and current of 150 mA, with stability of ±0.005%. The test samples consisted of powdered specimens obtained from core drilling, which were compressed into pellets using a hydraulic press before analysis.

#### 2.4.4. Scanning Electron Microscopy (SEM) Analysis of Samples

The bulk samples underwent gold sputtering using an EDT-2000 ion sputter coater (Beijing Vision Precision Instruments Technology Co., Ltd., Beijing, China). The sputtering parameters were set to a current of 10 mA, atmospheric pressure of 0.2 mbar (20 Pa), and sputtering duration of 90 s, resulting in a gold coating thickness of approximately 20 nm. Scanning electron microscopy (SEM) analysis was conducted using an EPMA-8050G field emission electron probe microanalyzer (Shimadzu Corporation, Kyoto, Japan), featuring a secondary electron image resolution of 3 nm, accelerating voltage of 30 kV, and beam current of 3 μA. The microstructural morphology of the gold-sputtered bulk samples was examined using this instrument.

## 3. Results and Discussion

### 3.1. Corrosion Failure States of Concrete Under Field Exposure in the Salt Lake

**Figure 2:**
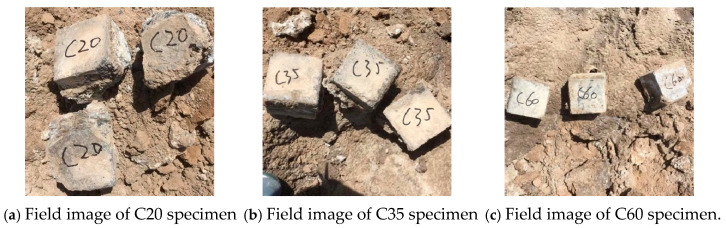
Field images of the concrete specimens at the Qarhan Salt Lake site.

Figure 2 presents field images of concrete specimens exposed at the Qarhan Salt Lake site. It is evident that the C20 concrete, which was OPC, had undergone considerable and severe deformation. A large amount of crystalline salts had accumulated on the specimen surface, leading to severe expansion, which in turn caused extensive spalling. Large corrosion pits had formed, and the specimen had completely lost its original cubic geometry. During handling, the C20 specimen displayed extreme brittleness, breaking apart with minimal contact. Given the extent of its damage, further testing on this specimen was not feasible. The C35 concrete, also OPC, had partial surface spalling. Compared to the C20 specimen, the degree of spalling was relatively less severe. Under the influence of the field corrosion environment, many corrosion pits formed on the surface of the C35 specimen, making its surface uneven. The C60 concrete, which was HPC, maintained an overall intact surface condition. The extent of corrosion pit formation owing to field exposure was limited, and the specimen surface remained relatively smooth.

In summary, based on the visual observation and analysis of the corrosion conditions of the field-exposed concrete specimens, it can be concluded that the HPC demonstrated superior corrosion resistance compared to OPC under the same exposure conditions. Additionally, a higher-strength grade corresponded to greater resistance to corrosion.

### 3.2. Analysis of Corrosion Products in Concrete of Different Strength Grades

#### 3.2.1. XRD and TG-DTA Analysis of C25 Concrete

**Figure 3:**
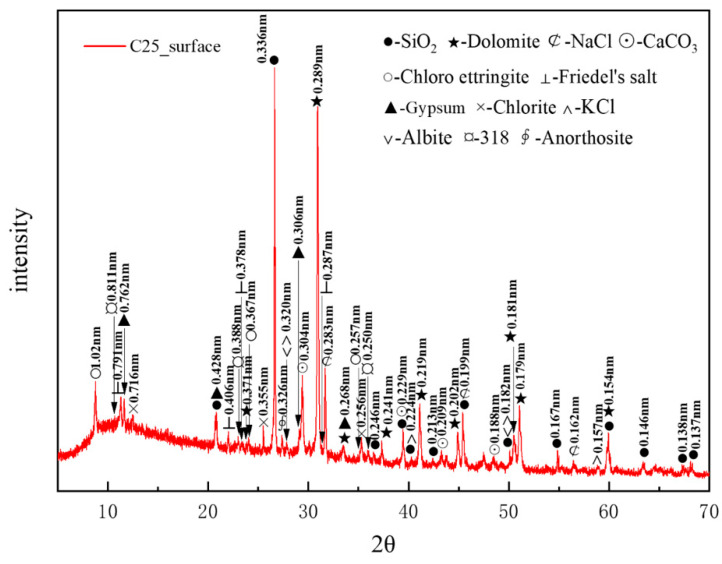
XRD pattern of the surface samples directly collected from the C25 specimen.

Figure 3 presents the XRD pattern of surface samples directly collected from the C25 specimen. The main crystalline phases identified included quartz (SiO_2_), with characteristic peaks at 0.336, 0.428, and 0.181 nm, and dolomite (CaMg(CO_3_)_2_), with characteristic peaks at 0.289, 0.219, and 0.179 nm. The quartz originated from the sand used as a raw material, whereas the dolomite was derived from the aggregate. Other phases included albite (characteristic peaks at 0.320, 0.242, and 0.182 nm), chlorite (characteristic peaks at 0.716, 0.355, and 0.256 nm), and anorthosite (characteristic peak at 0.326 nm), which were present as impurities in the sample. A minor presence of magnesium oxychloride hydrate 318 (3Mg(OH)_2_·MgCl_2_·8H_2_O) was detected, with characteristic peaks at 0.811, 0.388, and 0.250 nm. Calcium carbonate (CaCO_3_), with characteristic peaks at 0.304, 0.229, and 0.209 nm, was identified as a carbonation product. The chloride-induced corrosion products included sodium chloride (NaCl, characteristic peaks at 0.283, 0.199, and 0.160 nm), potassium chloride (KCl, characteristic peaks at 0.320, 0.224, and 0.157 nm), chloro-ettringite (3CaO·Al_2_O_3_·3CaCl_2_·30H_2_O, characteristic peaks at 1.02, 0.367, and 0.257 nm), and Friedel’s salt (3CaO·Al_2_O_3_·CaCl_2_·10H_2_O, characteristic peaks at 0.791, 0.406, and 0.287 nm). Among them, NaCl and KCl were physical crystallization products from the salt lake brine. The sulfate-induced corrosion product identified was gypsum (CaSO_4_·2H_2_O), with characteristic peaks at 0.762, 0.428, and 0.306 nm.

Based on the intensity of the characteristic peaks of each corrosion product, the main physicochemical corrosion products in the C25 concrete, ranked in order of decreasing content, were sodium chloride, calcium carbonate, chloro-ettringite, Friedel’s salt, and gypsum.

**Figure 4:**
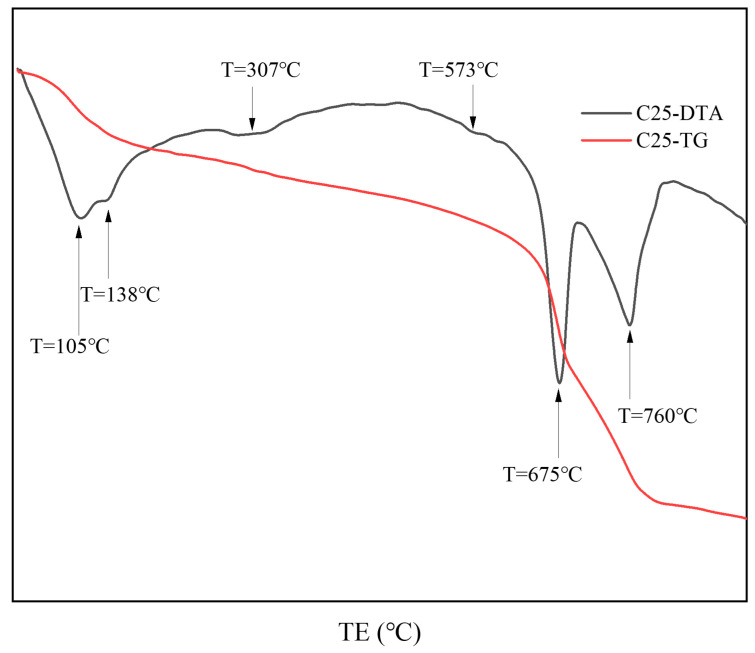
TG-DTA curves of surface samples directly collected from the C25 specimen.

Figure 4 presents the thermal analysis results of surface samples directly collected from the C25 specimen. Based on the XRD analysis, the results show that the endothermic trough at approximately 105 °C represents the loss of bound water from the chloro-ettringite and the dehydration of dihydrate gypsum to form hemihydrate gypsum (CaSO_4_·1/2H_2_O) [10,11]. The endothermic trough at approximately 138 °C corresponds to the decomposition of hemihydrate gypsum into anhydrous calcium sulfate (CaSO_4_) [10,11], as well as the loss of four interlayer water molecules from Friedel’s salt [12,13]. In the 200–400 °C range, the main thermal event corresponds to the release of six structural water molecules from Friedel’s salt, a feature commonly used to identify its presence in cement pastes or mortars [13]. The endothermic trough at 307 °C specifically corresponds to the endothermic dehydration of Friedel’s salt. At 573 °C, an endothermic trough was evident, indicating the phase change in quartz [10]. Within the 600–800 °C range, a major endothermic event was evident, corresponding to the stepwise thermal decomposition of dolomite in the specimen [14]. Initially, at approximately 675 °C, the decomposition of magnesium carbonate produced calcium carbonate and magnesium oxide, corresponding to the decomposition of magnesium carbonate. Next, at approximately 760 °C, both the calcium carbonate formed from dolomite and the carbonation-induced calcium carbonate underwent thermal decomposition [15]. The thermal analysis results for the C25 sample were consistent with the findings from the XRD analysis.

#### 3.2.2. XRD and Thermogravimetric–Differential Thermal Analysis (TG-DTA) of C60 Concrete

**Figure 5:**
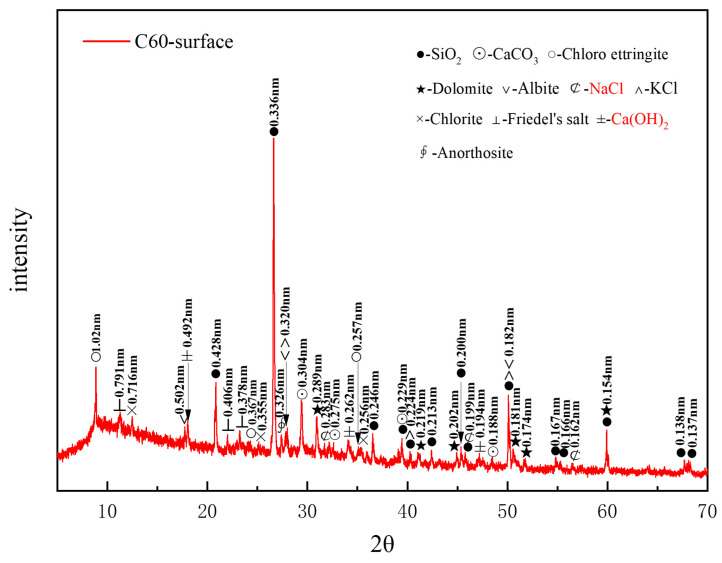
XRD pattern of the surface samples directly collected from the C60 specimen.

Figure 5 presents the XRD pattern of surface samples directly collected from the C60 specimen. A considerable amount of quartz in the C60 originated from the sand used as a raw material, whereas the dolomite was derived from the aggregate. Albite, chlorite, and anorthosite were present as impurities, and calcium carbonate was also detected in the C60. The identified corrosion products included chloro-ettringite, Friedel’s salt, sodium chloride, and potassium chloride. The characteristic peaks of calcium hydroxide (Ca(OH)_2_), a hydration product of cement, could be observed at 0.262, 0.492, and 0.194 nm. Based on the intensity of the characteristic peaks of each corrosion product, the main physicochemical corrosion products in the C60 concrete, ranked in order of decreasing content, were chloro-ettringite, Friedel’s salt, sodium chloride, and potassium chloride. Compared with the C25, the C60 lacked the 318 phase and gypsum, contained a lower amount of dolomite, and had considerably less sodium chloride. Moreover, calcium hydroxide, which was absent in the C25, appeared in the C60.

**Figure 6:**
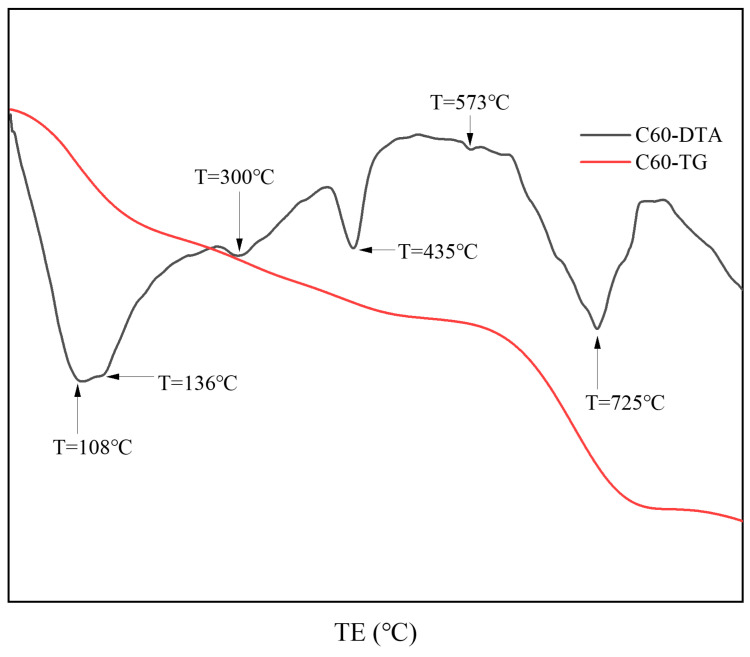
TG-DTA curves of the surface samples directly collected from the C60 specimen.

Figure 6 presents the thermal analysis results from the surface samples directly collected from the C25 specimen. Based on the XRD analysis, the endothermic peak at 108 °C corresponded to the dehydration of calcium silicate hydrate (C-S-H) and chloro-ettringite. The peak at 136 °C represented the loss of four interlayer water molecules from Friedel’s salt, whereas the peak at 300 °C corresponded to the loss of six interlayer water molecules from Friedel’s salt. The endothermic peak at 435 °C could be attributed to the dehydration of calcium hydroxide, whereas the endothermic trough at 573 °C corresponded to the phase transition of quartz. Finally, the peak at 725 °C represented the endothermic decomposition of calcium carbonate. These thermal analysis results were consistent with the XRD findings.

### 3.3. Depth Distribution Law of Corrosion Products in Concrete

**Figure 7:**
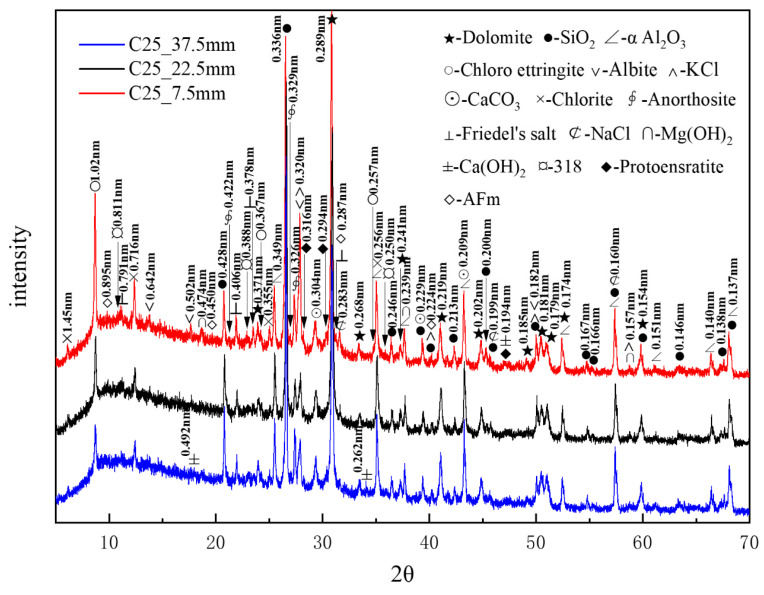
XRD patterns of the C25 samples at different depths obtained through core drilling sampling.

Figure 7 presents the XRD patterns of the C25 samples at different depths obtained through core drilling sampling. Compared to the surface samples obtained by direct sampling, the phase composition of the core-drilled samples had slight differences, which could be attributed to variations in the sampling methods and locations. α-aluminum oxide (α-Al_2_O_3_), with characteristic peaks at 0.256, 0.160, and 0.209 nm, was added as an internal standard at 20% by mass. A considerable amount of quartz and dolomite originated from the raw materials, whereas relatively high amounts of albite, chlorite, and anorthosite were present as impurities. Trace amounts of protoenstatite—identified as an impurity in the sample—were characterized by peaks at 0.316, 0.194, and 0.294 nm. Chloro-ettringite was the main corrosion product. It appeared abundantly in the shallow layers, yet its content decreased gradually with depth. The content of corrosion products sodium chloride, potassium chloride, and Friedel’s salt also decreased with increasing depth. By contrast, the content of calcium carbonate did not change noticeably with depth. Trace amounts of brucite (Mg(OH)_2_)—identified by characteristic peaks at 0.239, 0.474, and 0.157 nm, along with the 318 phase and AFm (3CaO·Al_2_O_3_·CaSO_4_·12H_2_O), with characteristic peaks at 0.895, 0.450, and 0.287 nm—appeared in the shallow layers but disappeared in the deeper layers, following a decreasing trend with depth. By contrast, calcium hydroxide was only detected in the deeper layers, indicating an increase in calcium hydroxide content with depth. XRD analysis confirmed that the major corrosion products, in descending order of content, were chloro-ettringite, calcium carbonate, sodium chloride, Friedel’s salt, brucite, the 318 phase, and AFm.

**Figure 8:**
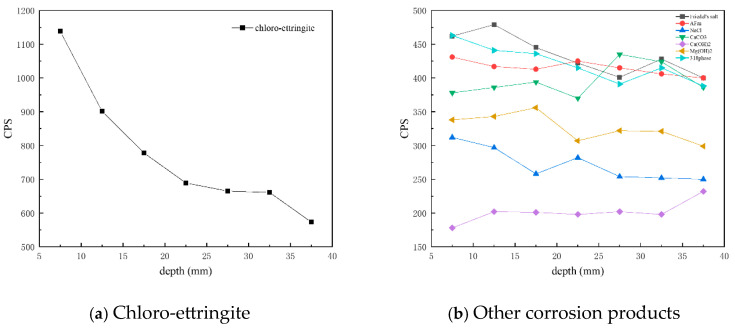
CPS plots of different phases in the C25 samples at different depths obtained through core drilling sampling.

Figure 8 presents CPS plots based on the peak intensity data extracted from XRD analysis of the C25 samples at different depths. It is evident that the content of chloro-ettringite—the main corrosion product—decreased with increasing depth. Similarly, the content of Friedel’s salt, AFm, the 318 phase, brucite, and sodium chloride also decreased with depth, whereas that of calcium carbonate and calcium hydroxide increased with depth.

**Figure 9:**
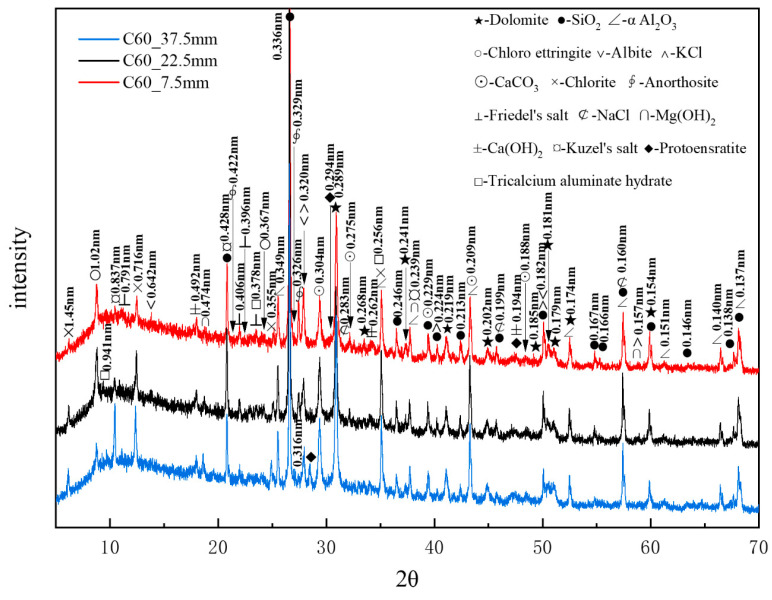
XRD patterns of the C60 samples at different depths obtained through core drilling sampling.

Figure 9 presents the XRD patterns of the C60 samples at different depths obtained through core drilling sampling, with α-aluminum oxide being used as an internal standard. A considerable amount of quartz and dolomite originated from the raw materials, whereas relatively high amounts of albite, chlorite, and anorthosite were present as impurities. Trace amounts of protoenstatite were also identified as an impurity. Chloro-ettringite was more abundant in the shallow layers than in the deep layers, its content decreasing with depth. By contrast, Kuzel’s salt (3CaO·Al_2_O_3_·1/2CaCl_2_·1/2CaSO_4_·10H_2_O), with characteristic peaks at 0.837, 0.428, and 0.239 nm, was present in smaller amounts in the shallow layers than in the deep layers, its content increasing with depth. The potassium chloride content decreased with increasing depth, whereas that of brucite increased with depth. Trace amounts of sodium chloride and Friedel’s salt decreased with depth. The hydration product calcium hydroxide appeared in the shallow layers but exhibited no major variation overall. Additionally, trace amounts of tricalcium aluminate hydrate (3CaO·Al_2_O_3_·3CaCO_3_·32H_2_O), with characteristic peaks at 0.941, 0.256, and 0.378 nm, appeared in the deeper layers. The increase in calcium carbonate content with depth could be attributed to the presence of limestone, the active mineral additive in the cement.

XRD analysis confirmed that the main corrosion products, in descending order of content, were chloro-ettringite, Kuzel’s salt, Friedel’s salt, brucite, and sodium chloride. Compared with the XRD patterns of the C25, it is evident that the C60 contained considerably less chloro-ettringite, though both concrete samples exhibited a similar depth-dependent variation in chloro-ettringite content. Calcium hydroxide appeared in the surface layers of the C60 but it had obvious peaks only in the deeper layers of the C25. The diffraction peaks of brucite in the C60 were more obvious than those in the C25, whereas the diffraction peaks of sodium chloride in the C60 were notably weaker than those in the C25.

**Figure 10:**
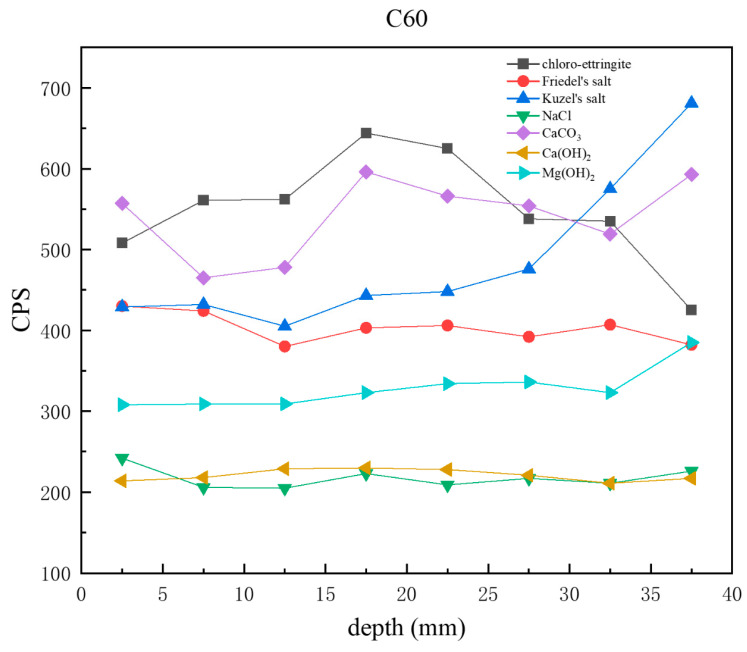
CPS plots of different phases in the C60 samples at different depths obtained through core drilling sampling.

Figure 10 presents CPS plots based on the peak intensity data extracted from XRD analysis of the C60 samples at different depths. Unlike the C25, in which chloro-ettringite was the dominant corrosion product, the C60 contained relatively lower amounts of chloro-ettringite. In the C60, the content of both chloro-ettringite and Friedel’s salt showed a decreasing trend with depth, whereas the content of Kuzel’s salt increased with depth. In the shallow layers, chloro-ettringite and Friedel’s salt were more abundant, whereas Kuzel’s salt was less prevalent. In the deeper layers, the content of Kuzel’s salt was high, whereas that of chloro-ettringite and Friedel’s salt was low. Compared with the C25, the C60 contained lower amounts of sodium chloride, which decreased with depth. The calcium hydroxide content in the C60 remained relatively uniform, whereas the brucite content increased with depth. The overall calcium carbonate content was relatively high.

### 3.4. Correlation Between Concrete Corrosion Products and Strength Grade

**Figure 11:**
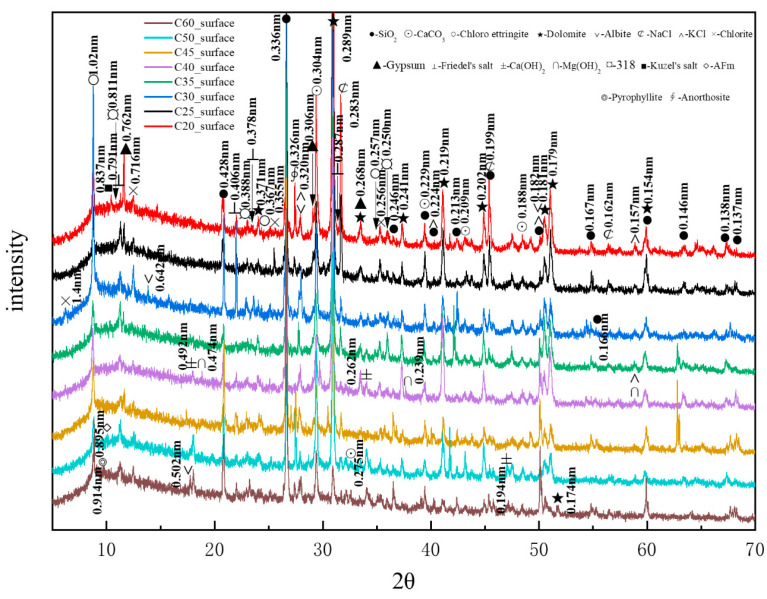
XRD patterns of the surface samples of different strength grades obtained through direct sampling.

Figure 11 presents the XRD patterns of the surface samples of different strength grades obtained through direct sampling. It is evident that a large amount of quartz and dolomite originated from the main raw materials, whereas albite, chlorite, anorthosite, and pyrophyllite (Al_2_Si_4_O_10_(OH)_2_, with a characteristic peak at 0.914 nm, were impurities within the raw materials. The hydration product calcium hydroxide could be observed in higher-strength samples. The identified physicochemical corrosion products included calcium carbonate, chloro-ettringite, sodium chloride, gypsum, Friedel’s salt, potassium chloride, brucite, the 318 phase, AFm, and Kuzel’s salt, with the content of each corrosion product varying among samples of different strength grades. The XRD patterns generally showed a decreasing trend in the content of corrosion products as the strength grade increased. The mechanical strength of concrete is influenced by its bulk density. Higher bulk density corresponds to increased concrete compactness, resulting in enhanced mechanical strength. Denser concrete microstructures effectively resist the penetration of corrosive ions, thereby reducing corrosion depth.

**Figure 12:**
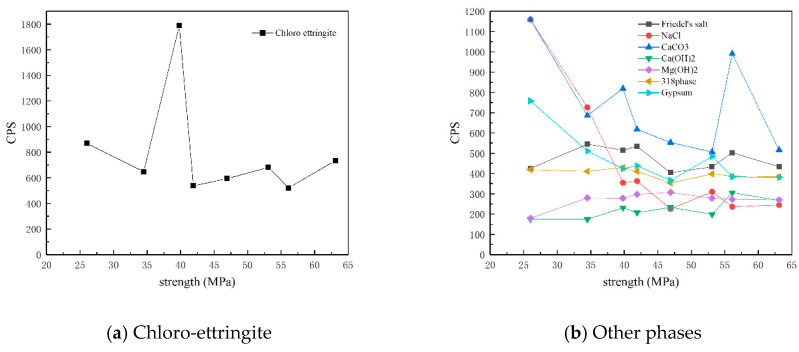
CPS plots of different phases in the surface samples of different strength grades obtained through direct sampling.

Figure 12 shows the CPS values of different phases in the surface samples of different strength grades obtained through direct sampling. It is evident that the overall content of physicochemical corrosion products—including chloro-ettringite, Friedel’s salt, sodium chloride, calcium carbonate, gypsum, and the 318 phase—decreased as the strength grade increased. By contrast, the content of the hydration product calcium hydroxide increased with strength grade.

**Figure 13:**
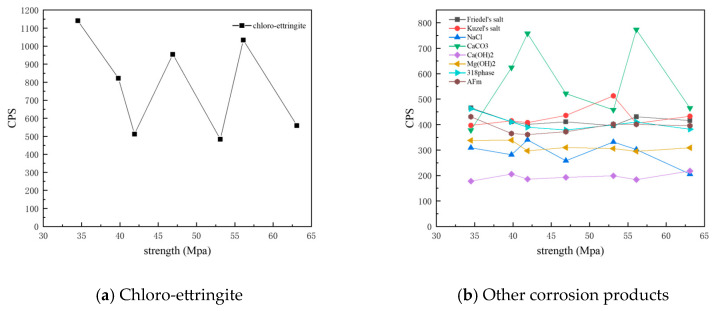
CPS plots of different phases in the samples of different strength grades obtained through core drilling sampling at a depth of 7.5 mm.

Figure 13 shows the CPS values of different phases in the surface samples of different strength grades obtained through core drilling sampling at a depth of 7.5 mm. It is evident that the overall content of physicochemical corrosion products—including chloro-ettringite, Friedel’s salt, sodium chloride, calcium carbonate, AFm, and the 318 phase—decreased as the strength grade increased. By contrast, the content of the hydration product calcium hydroxide increased with strength grade. The corrosion product Kuzel’s salt exhibited an increasing trend with higher-strength grades.

### 3.5. Ion Content Distribution in Concrete and Its Influence on Physicochemical Corrosion Products

#### 3.5.1. Chloride Ion Distribution and Chloride Corrosion Products

**Figure 14:**
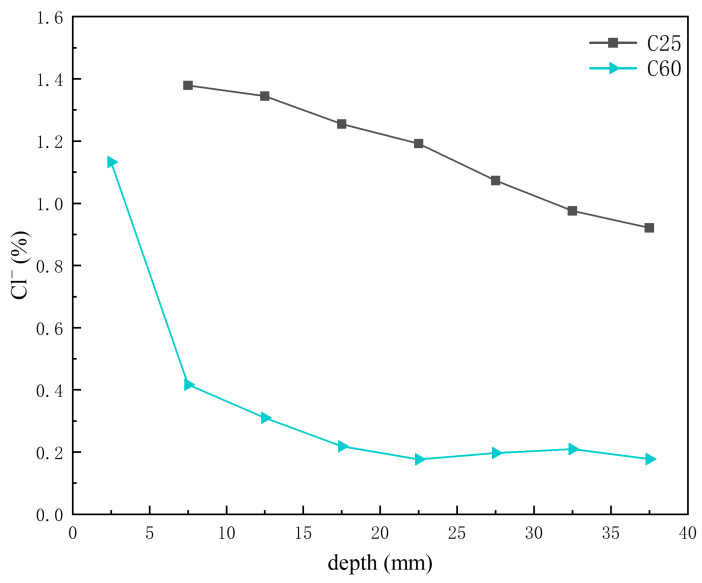
Depth-dependent variation in chloride ion content of C25 and C60 core drilling samples.

Previous analyses indicated that the main chloride-induced corrosion products in the C25 were Friedel’s salt, chloro-ettringite, the 318 phase, potassium chloride, and sodium chloride, whereas in the C60, the main chloride-induced corrosion products were Kuzel’s salt, Friedel’s salt, chloro-ettringite, potassium chloride, and sodium chloride. Figure 14 shows the depth-dependent variation in chloride ion content obtained through XRF analysis of core drilling samples from the C25 and C60 specimens. The chloride ion content in the C25 was considerably higher than that in the C60, and both showed a decreasing trend with depth. Li Wanjin [16] and Li Lin [17] demonstrated through numerical simulations and immersion experiments, respectively, that chloride ion concentration decreases with greater penetration depth, peaking at the concrete surface and diminishing toward the interior. Comparing this trend with the CPS plots above, the content of Friedel’s salt, chloro-ettringite, the 318 phase, potassium chloride, and sodium chloride all decreased with decreasing chloride ion concentration. However, the formation of Kuzel’s salt increased as the chloride ion content decreased, suggesting that Kuzel’s salt preferentially formed under conditions of insufficient chloride availability. The chloro-ettringite content was the main factor contributing to the difference in chloride ion content between the C25 and C60.

#### 3.5.2. Sulfate Ion Distribution and Sulfate Corrosion Products

**Figure 15:**
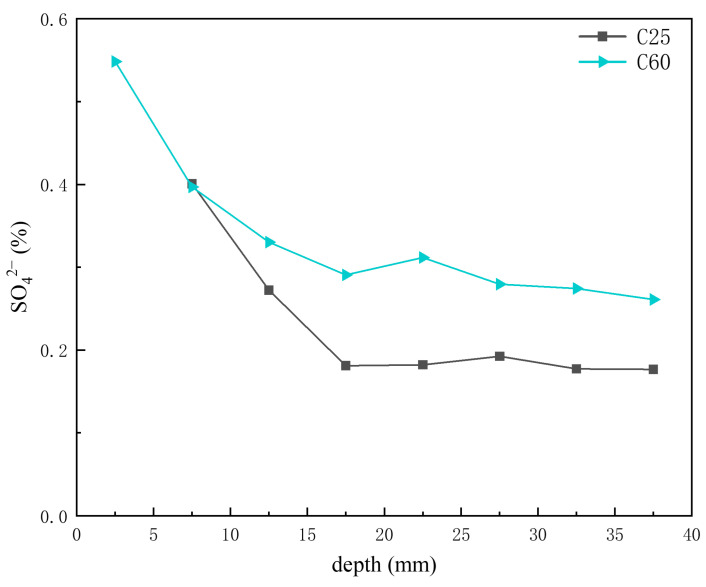
Depth-dependent variation in the sulfate ion content of the C25 and C60 core drilling samples.

Based on the previous analysis of corrosion product content at different depths, AFm was present in trace amounts in the C25, whereas Kuzel’s salt was more abundant in the C60. Figure 15 presents the depth-dependent variation in sulfate ion content in the core drilling samples. The sulfate ion content in the C60 was slightly higher than that in the C25, but the difference was not as obvious as that observed for chloride ions. The AFm content in the C25 decreased with increasing depth, whereas the Kuzel salt content in the C60 increased. Given that sulfate ions in Kuzel’s salt were partially substituted by chloride ions, the sulfate ion content in Kuzel’s salt was lower than that in AFm at the same molecular weight. Consequently, an increase in Kuzel’s salt corresponded to a decrease in sulfate ion content, which was consistent with the trend observed in Figure 15. Gao Rundong [18] validated through experimental investigations that sulfate ion concentrations exhibit a decreasing trend with increasing erosion depth, characterized by higher accumulation at the concrete surface and reduced penetration into the interior.

#### 3.5.3. Sodium Ion Distribution and Physical Chloride Corrosion Products

**Figure 16:**
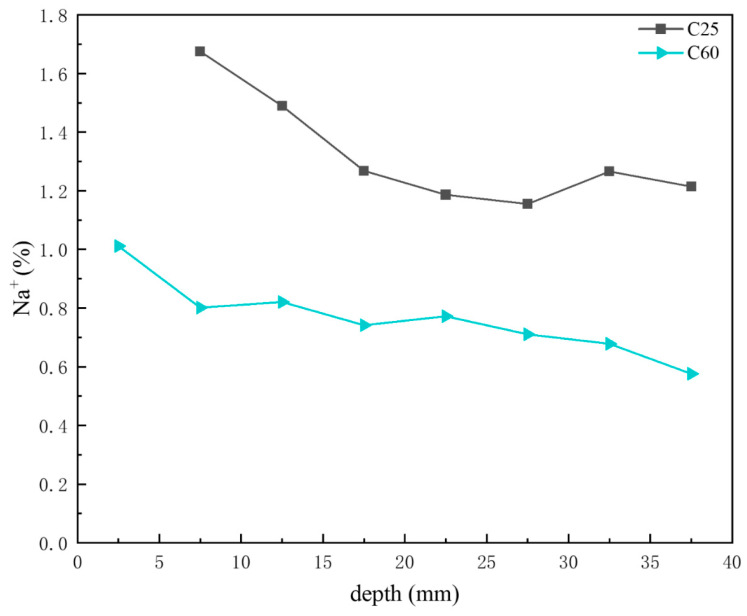
Depth-dependent variation in the sodium ion content of the C25 and C60 core drilling samples.

Figure 16 shows the depth-dependent variation in sodium ion content in core drilling samples. Based on the previous CPS analysis, the sodium chloride content in the C25 was generally higher than that in the C60, with both displaying a decreasing trend with depth. This trend was consistent with the sodium ion content distribution shown in Figure 16, where the sodium ion content in the C25 exceeded that in the C60, with both decreasing with increasing depth.

#### 3.5.4. Magnesium Ion Distribution and Magnesium Salt Corrosion Products

**Figure 17:**
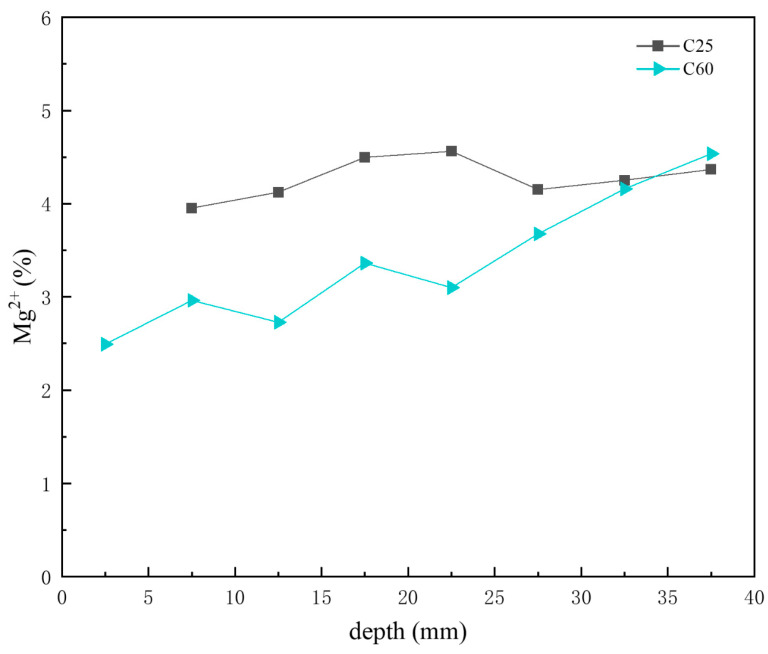
Depth-dependent variation in the magnesium ion content of the C25 and C60 core drilling samples.

Figure 17 presents the depth-dependent variation in magnesium ion content in the core drilling samples. Combined with the CPS analysis above, both the C25 and C60 had relatively low magnesium salt content. The variation trend of the magnesium ion content in the C25 was not significant. Moreover, in the C60, the magnesium ion content changed, increasing with depth. This trend, as shown in Figure 17, corresponds to variations in the brucite content observed in the CPS analysis for the C60. As the transformation of brucite into the 318 phase leads to an increase in magnesium ion content, the overall depth-dependent variation in the C25 is consistent with the trends shown in Figure 17.

### 3.6. Microstructural Analysis of Corroded Concrete

**Figure 18:**
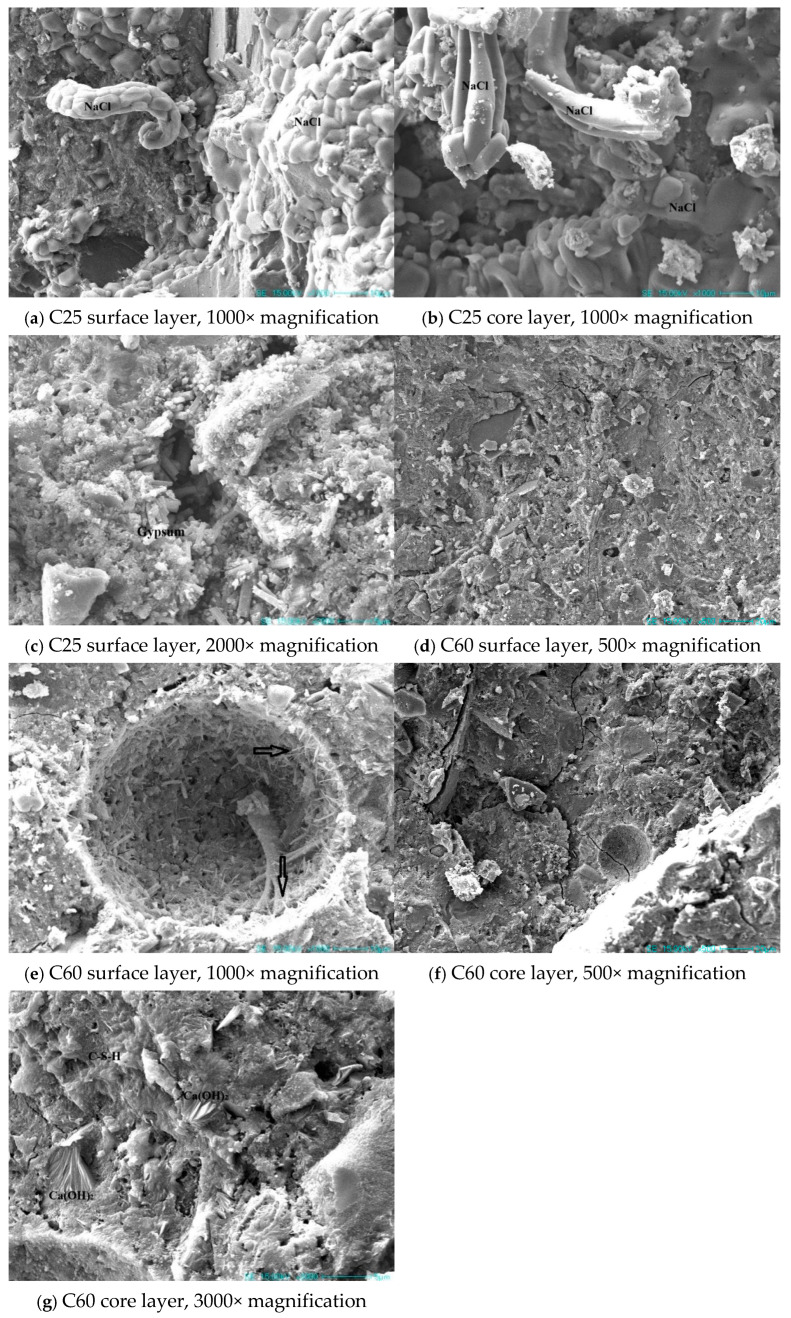
SEM images of the field-exposed concrete specimens from Qarhan Salt Lake.

Figure 18 presents SEM images of the concrete specimens with different strength grades. In the C25 specimen, a large amount of sodium chloride could be observed in both the surface and core layers. In the surface layer, sodium chloride was seen extruding from the pores. Sodium chloride was present in large quantities throughout the sample, causing physical crystallization damage to the concrete. Additionally, a substantial amount of rod-shaped gypsum was evident in the cracks of the surface layer. The expansion of this gypsum within the cracks led to volume expansion, causing further damage to the concrete.

The C60 specimen had a dense surface in both the surface and core layers, with little sodium chloride evident. Compared to the C25, the corrosion products on the surface layer of the C60 were considerably reduced, with only a small amount of needle-like deposits adhering to the pore walls. In the core layer, a considerable amount of flaky calcium hydroxide was evident. Based on prior observations, the C25 had obvious gypsum and sodium chloride phases. The presence of chloro-ettringite was particularly apparent in samples at varying depths. This was consistent with the abundant sodium chloride in the microstructure, indicating a high chloride ion content. In the C60, calcium hydroxide was identified in previous analyses, and its presence was further confirmed using the microstructural images, which showed a substantial amount of flaky calcium hydroxide in the core layer. Sodium chloride was rarely detected in the C60, consistent with the previously observed difference in the sodium and chloride ion content between the C25 and C60. This also indirectly suggests that the C60 contained lower amounts of chloro-ettringite and Friedel’s salt compared to the C25.

### 3.7. Long-Term Physicochemical Corrosion of Concrete in the Qarhan Salt Lake Environment

Firstly, calcium hydroxide (a hydration product of cement) undergoes carbonation to form calcium carbonate.

Ca(OH)_2_ + CO_2_ + H_2_O → CaCO_3_ + 2H_2_O (1)

According to studies by Lingyu et al. [8] and Yu Hongfa [10], the following chemical reactions may occur in the Qinghai Salt Lake environment:

C_3_AH_6_ + Ca^2+^ + 2Cl^−^ + 4H_2_O → 3CaO•Al_2_O_3_•CaCl_2_•10H_2_O (2)

C_3_S_2_H_3_ + 3Mg^2+^ + 3SO_4_^2−^ + 8H_2_O → 3[CaSO_4_•2H_2_O] + 3Mg(OH)_2_ + 2[SiO_2_•H_2_O] (3)

3CaO•Al_2_O_3_•3CaSO_4_•32H_2_O(AFt) → 3CaO•Al_2_O_3_•CaSO_4_•12H_2_O(AFm) + 2[CaSO_4_•2H_2_O] + 16H_2_O (4)

3CaO•Al_2_O_3_•3CaSO_4_•32H_2_O(AFt) + Ca^2+^ + 2Cl^−^ → 3CaO•Al_2_O_3_•CaCl_2_•10H_2_O + 3[CaSO_4_•2H_2_O] + 16H_2_O (5)

3CaO•Al_2_O_3_•3CaSO_4_•32H_2_O(AFt) + 6Cl^−^ → 3CaO•Al_2_O_3_•3CaCl_2_•30H_2_O + 3SO_4_^2−^ + 2H_2_O (6)

Ca(OH)_2_ + Mg^2+^ + 2Cl^−^ + H_2_O → CaCl_2_ + Mg(OH)_2_ + H_2_O (7)

Ca(OH)_2_ + Mg^2+^ + SO_4_^2−^ + H_2_O → CaSO_4_•2H_2_O + Mg(OH)_2_ (8)

3CaO•Al_2_O_3_•CaSO_4_•12H_2_O(AFm) + Cl^−^ → 3CaO•Al_2_O_3_•1/2CaSO_4_•1/2 CaCl_2_•10H_2_O + 1/2SO_4_^2−^ + 2H_2_O (9)

3CaO•Al_2_O_3_•CaSO_4_•12H_2_O(AFm) + 2Cl^−^ → 3CaO•Al_2_O_3_•CaCl_2_•10H_2_O + SO_4_^2−^ + 2H_2_O (10)

3Mg(OH)_2_ + MgCl_2_ + 8H_2_O → 3Mg(OH)_2_•MgCl_2_•8H_2_O (11)

No ettringite was detected in either the C25 or C60, indicating that ettringite gradually decomposed in the chloride-rich salt lake solution and transformed into chloride-induced corrosion products. The corrosion reactions (4)–(6) describe the decomposition of ettringite into AFm, Friedel’s salt, and chloro-ettringite, respectively. The corrosion reactions (9) and (10) illustrate the transformation of AFm into Kuzel’s salt and Friedel’s salt owing to chloride attack. Based on previous analyses, the C25 contained a considerable amount of chloro-ettringite, a relatively high content of Friedel’s salt, and a small amount of AFm, alongside a higher sodium chloride content compared to the C60. The C60 had a lower chloro-ettringite content but contained Friedel’s salt and Kuzel’s salt. However, the C60 had less chloro-ettringite than the C25. In the C60, the amounts of chloro-ettringite and Friedel’s salt decreased with depth, whereas Kuzel’s salt increased.

These findings indicate that the transformation of ettringite into chloride-induced corrosion products was closely related to the internal chloride ion concentration in the concrete. As the chloride ion concentration increased, the transformation sequence of ettringite into chloride corrosion products occurred in the following order: AFm → Kuzel’s salt → Friedel’s salt → chloro-ettringite. The formation of gypsum could be attributed to reactions (3), (4), (5), and (8). These corrosion reactions consumed hydration products (such as C-S-H, ettringite, and calcium hydroxide), leading to the disintegration of the hardened concrete structure. This was consistent with the SEM observations of gypsum in the C25 surface sample, where neither ettringite nor calcium hydroxide was detected. Reactions (3), (7), and (8) describe the conversion of the main cement hydration products (such as C-S-H and calcium hydroxide) into brucite owing to corrosion.

The formation of the 318 phase followed Reaction (11). This process consumed brucite while increasing the concentration of magnesium ions. This was consistent with the observation of trace amounts of 318 phase and brucite in the C25, whereas only brucite was detected in the C60. Additionally, the C60 contained more brucite than the C25. Consequently, under salt lake conditions, the formation sequence of magnesium salt corrosion products in concrete occurred in the following order: MH → 318 phase.

According to Yu Hongfa [10], field-exposed specimens in the salt lake environment experienced low-temperature chemical corrosion. It is evident from the photos taken at the salt lake site that the C20 concrete specimen suffered severe physical crystallization corrosion, resulting in a large amount of salt crystallization on its surface, significant spalling of the concrete, and a complete loss of its cubic shape. SEM images further confirmed the formation of large amounts of sodium chloride and other physical crystallization products. Chemical corrosion was driven primarily by the formation of AFm, Kuzel’s salt, Friedel’s salt, and chloro-ettringite.

## 4. Conclusions

1. The concrete corrosion products varied depending on the concrete strength grade. The C25 saw the formation of gypsum, AFm, and magnesium oxychloride hydrate 318, whereas the C60 saw the formation of Kuzel’s salt.
2. For the same corrosion products, their content differed based on the concrete strength grade. In the C25, chloro-ettringite, sodium chloride, and Friedel’s salt were more abundant, whereas calcium carbonate and brucite were less prevalent. By contrast, the C60 contained lower amounts of chloro-ettringite, sodium chloride, and Friedel’s salt but had higher quantities of calcium carbonate and brucite.
3. The distribution of corrosion products varied with depth within the same concrete strength grade. In the C25, the chloro-ettringite, Friedel’s salt, AFm, sodium chloride, 318 phase, and brucite content decreased with depth, whereas the calcium carbonate content increased. In the C60, the chloro-ettringite, Friedel’s salt, and sodium chloride content decreased with depth, whereas that of brucite and Kuzel’s salt increased. The concentrations of chloride, sulfate, and sodium ions exhibit a decreasing trend with increasing erosion depth in concrete, characterized by higher accumulation at the surface and reduced penetration into the interior. In contrast, magnesium ion concentrations demonstrate an inverse distribution pattern, progressively increasing with depth and forming distinctly different ionic penetration gradients.
4. A transformation phenomenon could be observed between the sulfate-induced and chloride-induced corrosion products, which was closely related to the internal chloride ion concentration of the concrete. As the chloride ion concentration increased, the transformation sequence of the sulfate corrosion products occurred in the following order: AFm → Kuzel’s salt → Friedel’s salt → chloro-ettringite.
5. Under salt lake conditions, the HPC exhibited superior corrosion resistance compared to the OPC. Moreover, the corrosion resistance improved with increasing concrete strength.

The scope of this research is limited to analyzing 8-year field-exposed OPC and HPC corrosion patterns in the Qarhan Salt Lake and elucidating the transformation mechanisms between sulfate and chloride corrosion products. Critical aspects such as long-term corrosion evolution throughout their service life and the quantitative impact of chloride ion concentrations on sulfate product transformation remain unexplored. Subsequent investigations may prioritize extended field exposure durations (e.g., 15–30 years) and systematic evaluation of chloride concentration effects on sulfate corrosion dynamics.

## Figures and Tables

**Table 1:** Physical and mechanical properties of the cement.

Model	Fineness	Specific Surface Area/m^2^ kg^−1^	Standard Consistency Water Requirement/%	Coagulation Time/h		Bending Strength/MPa		Compressive Strength/MPa	
				Initial Setting/d	Final Setting/d	3 d	28 d	3 d	28 d
P.II 52.5	0.8	412	25	1: 35	2: 26	5.6	9.3	26.8	57.2
P.O 42.5	0.8	348	26	2: 25	3: 40	5.5	7.6	21.6	48.7

**Table 2:** Chemical composition of the main raw materials (%).

Material	SiO_2_	Al_2_O_3_	CaO	MgO	SO_3_	Fe_2_O_3_	Na_2_O	K_2_O	Cl^−^	I.L
P.II 52.5 cement	19.56	3.78	65.88	2.42	2.41	3.69	0.50	0.82	0.022	0.94
P.O 42.5 cement	20.98	9.38	59.45	2.00	2.44	3.64	0.24	0.59	0.023	1.28
Grinding slag	26.09	26.88	37.38	5.6	1.75	0.67	0.49	0.83	0.014	0.31

**Table 3:** Physical and mechanical properties of the slag.

Material	Density/g·m^−5^	Specific Surface Area/m^2^·kg^−1^	Total Activity/%	Mobility Ratio/%
S95 grade mineral powder	26.09	26.88	37.38	5.6

**Table 4:** Physical properties and chemical composition of the aggregate.

Aggregate	Apparent Density/kg·m^−3^	Stacking Density/kg·m^−3^	Void Ratio/%	Sediment Percentage/%	Needle-like Particle Content/%	Crushing Index/%	SO42^−/^%	Cl^−/^%
Fine aggregate	2647	1472	38.1	5.6	-	-	0.11	0.0054
Coarse aggregate	2780	1525	41.0	0.8	2.9	8.2	0.02	0.0063

**Table 5:** Mix proportion of the concrete specimens.

Type	Material/kg m^−3^						Water–Cement Ratio	28 d Compressive Strength/MPa
	Cement	Slag	Sand	Stone	Water	Water Reducer		
C20	338		605	1102	195	5.41	0.58	26.0
C25	348	70	630	1114	193	5.56	0.55	34.5
C30	355	82	676	1130	195	6.99	0.45	39.8
C35	386	98	643	1143	197	7.74	0.41	41.9
C40	398	105	649	1155	199	8.55	0.40	46.9
C45	456	105	622	1156	196	9.53	0.35	53.1
C50	468	120	626	1162	192	9.99	0.33	56.1
C60	480	135	631	1168	192	12.3	0.31	63.1

**Table 6:** Chemical composition of the topsoil of strong saline soil in the Qarhan Salt Lake (%).

Chemical Composition	K^+^	Na^+^	Mg^2+^	Ca^2+^	Cl^−^	SO_4_^2−^	CO_3_^2−^	Total
Mass/%	0.08	10.04	0.50	3.66	17.88	0.70	4.31	37.17

## Data Availability

The original contributions presented in the study are included in the article, further inquiries can be directed to the corresponding authors.

## Abbreviations

The following abbreviations are used in this manuscript:

EDS	Energy-dispersive spectroscopy
HPC	High-performance concrete
OPC	Ordinary Portland concrete
SEM	Scanning electron microscopy
TG-DTA	Thermogravimetric–differential thermal analysis
XRD	X-ray diffraction
XRF	X-ray fluorescence
